# Efficient and generalizable cross-patient epileptic seizure detection through a spiking neural network

**DOI:** 10.3389/fnins.2023.1303564

**Published:** 2024-01-10

**Authors:** Zongpeng Zhang, Mingqing Xiao, Taoyun Ji, Yuwu Jiang, Tong Lin, Xiaohua Zhou, Zhouchen Lin

**Affiliations:** ^1^Department of Biostatistics, School of Public Health, Peking University, Beijing, China; ^2^National Key Lab of General AI, School of Intelligence Science and Technology, Peking University, Beijing, China; ^3^Department of Pediatrics, Peking University First Hospital, Beijing, China; ^4^Beijing International Center for Mathematical Research, Peking University, Beijing, China; ^5^Peking University Chongqing Institute for Big Data, Chongqing, China; ^6^Institute for Artificial Intelligence, Peking University, Beijing, China

**Keywords:** epilepsy, seizure detection, cross-patient, EEG, spiking neural network

## Abstract

**Introduction:**

Epilepsy is a global chronic disease that brings pain and inconvenience to patients, and an electroencephalogram (EEG) is the main analytical tool. For clinical aid that can be applied to any patient, an automatic cross-patient epilepsy seizure detection algorithm is of great significance. Spiking neural networks (SNNs) are modeled on biological neurons and are energy-efficient on neuromorphic hardware, which can be expected to better handle brain signals and benefit real-world, low-power applications. However, automatic epilepsy seizure detection rarely considers SNNs.

**Methods:**

In this article, we have explored SNNs for cross-patient seizure detection and discovered that SNNs can achieve comparable state-of-the-art performance or a performance that is even better than artificial neural networks (ANNs). We propose an EEG-based spiking neural network (EESNN) with a recurrent spiking convolution structure, which may better take advantage of temporal and biological characteristics in EEG signals.

**Results:**

We extensively evaluate the performance of different SNN structures, training methods, and time settings, which builds a solid basis for understanding and evaluation of SNNs in seizure detection. Moreover, we show that our EESNN model can achieve energy reduction by several orders of magnitude compared with ANNs according to the theoretical estimation.

**Discussion:**

These results show the potential for building high-performance, low-power neuromorphic systems for seizure detection and also broaden real-world application scenarios of SNNs.

## 1 Introduction

**Figure 1:**
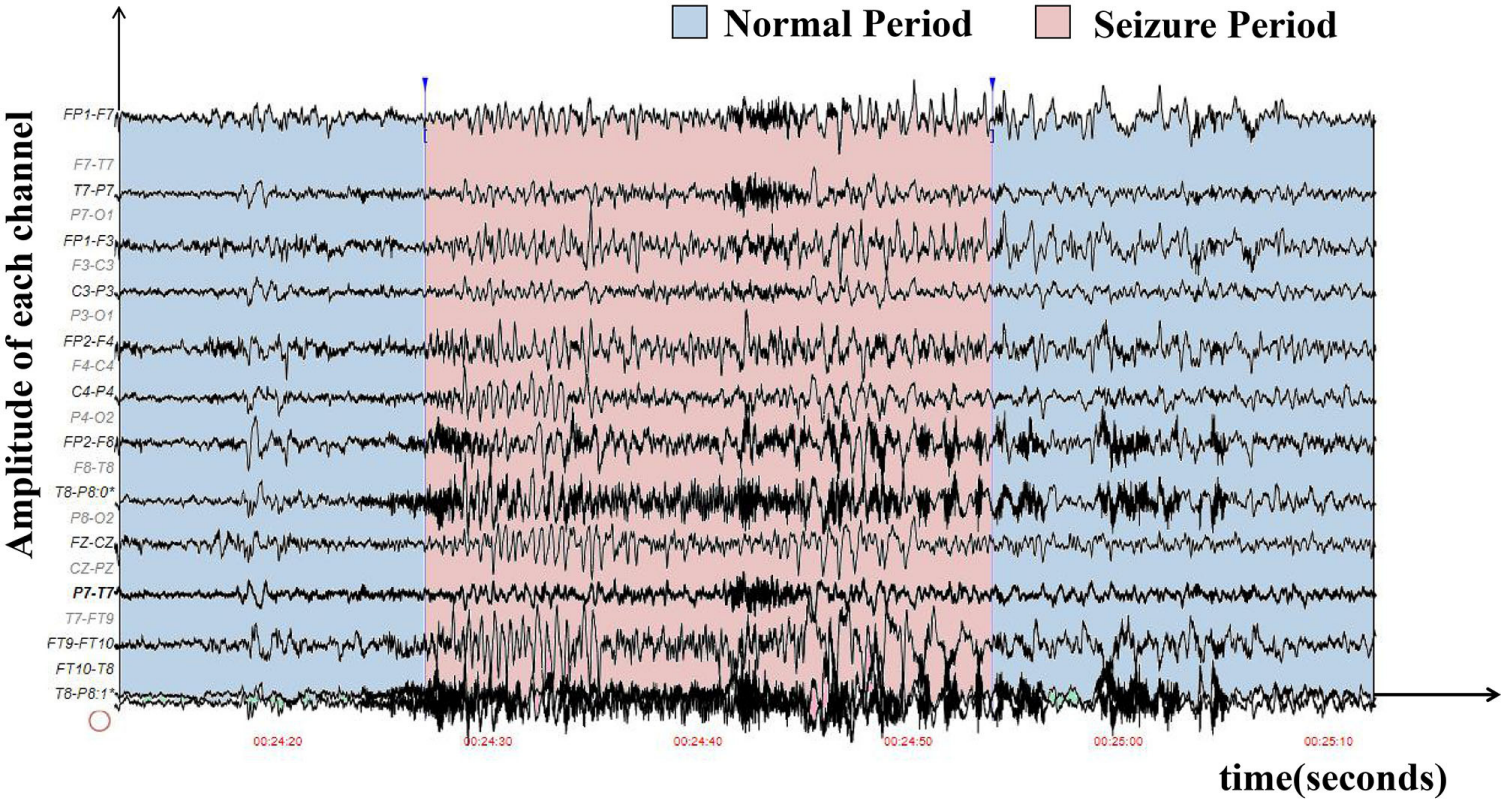
The waveform graph was sampled from the first patient in the CHB-MIT EEG dataset, where the x-axis represents time, and the y-axis represents the amplitude of each electrode. The seizure period is in red, while the normal period is in blue. It is difficult to distinguish seizure and non-seizure periods in manual diagnoses.

Epilepsy is caused by the abnormal firing of neurons in certain regions of the brain, and it has become the second most common disease of the nervous system (Mormann et al., 2007). It affects almost 50 million people around the world (World Health Organization, 2016). Automatic seizure detection can help with timely diagnosis and treatment, reducing the harm of epilepsy to patients, which is significant for both patients and doctors. The electroencephalogram (EEG) is the most commonly used analytical tool for clinical diagnosis of epilepsy by doctors (Ahmad et al., 2016). The hospitals often use the international 10-20 system placement method for the collection of an EEG (Cobb et al., 1958). As shown in Figure 1, the pattern of EEG signals is very complex and requires a lot of time and energy for professional doctors to make judgments. Thus, automatic epilepsy seizure detection, i.e., detecting one period of EEG signal whether to be on seizure automatically, is of great significance.

**Figure 2:**
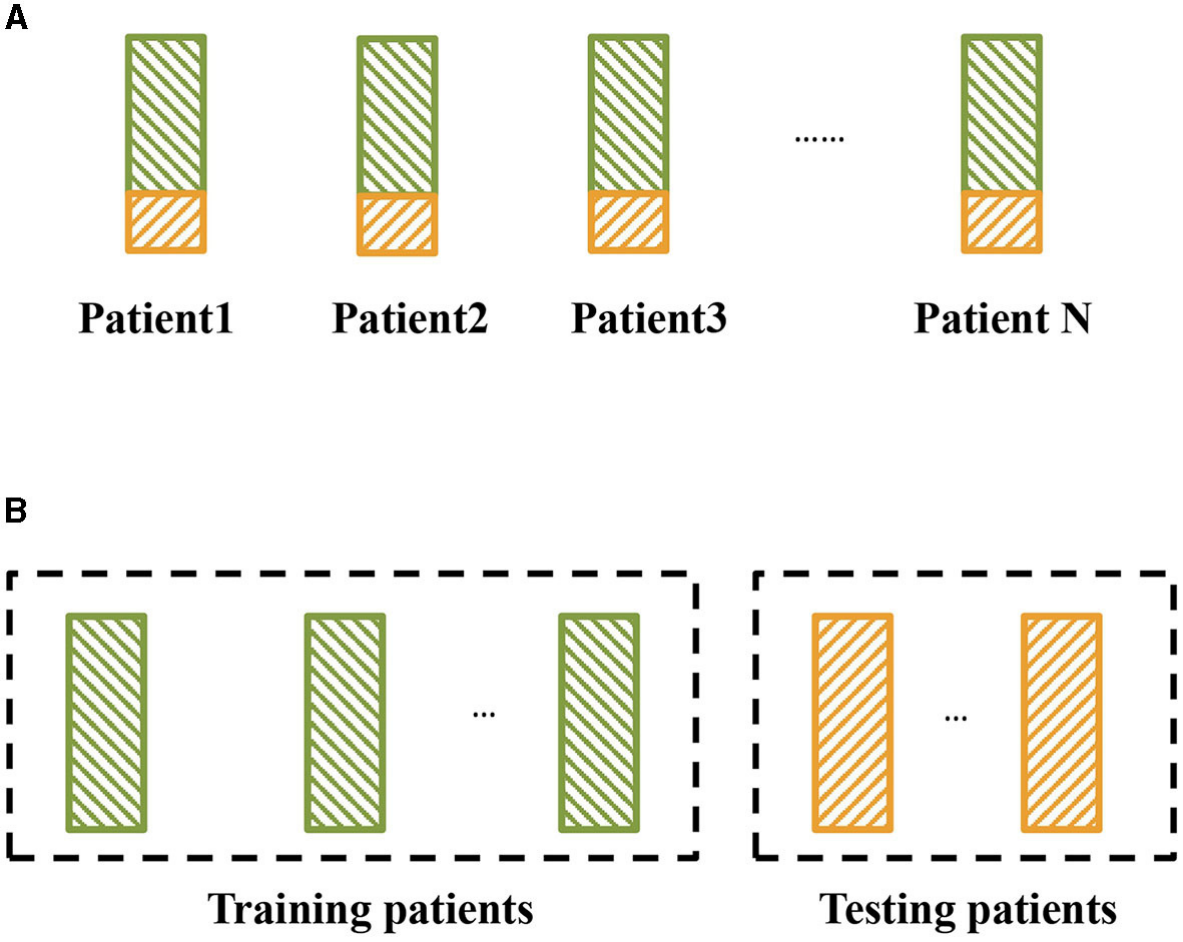
Two kinds of epileptic seizure detection settings. The green block represents the training set, and the yellow block represents the testing set. **(A)** Traditional setting for epileptic seizure detection, where the detection models are trained and tested on the same patients. **(B)** Cross-patient epileptic seizure detection, where the training patients and testing patients are different.

The mainstream seizure detection methods are based on deep learning with artificial neural networks (ANNs; Abdelhameed et al., 2018; Daoud and Bayoumi, 2019; Wei et al., 2019; Abiyev et al., 2020; Li et al., 2020; O'Shea et al., 2020; Ke et al., 2021, 2022; He et al., 2022; Shen et al., 2023). To achieve better performance, existing methods mostly treat EEG signals as image-like input, and thus they can learn from state-of-the-art computer vision models and techniques. However, existing ANN models ignore many of EEG's unique characteristics such as biological signal properties, which have much room to improve. Additionally, most existing seizure detection methods are patient-specific, while clinical applications need to consider cross-patient settings in practice. The difference between patient-specific algorithms and cross-patient algorithms is shown in Figure 2. The cross-patient seizure detection method can detect EEG signals belonging to any patient's brain and can be generalized for future patients. There are a few cross-patient seizure detection algorithms (Gómez et al., 2020; Peng et al., 2022; Tang et al., 2022; Zhao et al., 2022). They mainly improve deep learning with techniques such as data augmentation, feature disentanglement, adversarial optimization, etc. while still relying on common ANN models. Therefore, there is still much room for improvement considering models. We focus on improving cross-patient seizure detection with spiking neural network models.

As one of the existing bio-inspired models, spiking neural networks (SNNs) are based on modeling the dynamics of biological neurons and can be expected to better handle brain signals. Moreover, SNNs can be implemented on dedicated hardware, with high capacity and low energy cost (Akopyan et al., 2015; Davies et al., 2018; Pei et al., 2019; Sengupta et al., 2019). If considering portable monitoring equipment with low energy costs for endurance, SNNs can provide better support. On the other hand, SNNs are difficult to train and usually show a lacking performance compared with ANNs in some common tasks such as image classification (Rueckauer et al., 2017; Niu et al., 2023). It is still worth exploring what data/task is more suitable for SNNs to achieve better performance and how we can leverage the advantages of SNNs.

This paper focuses on the combination of cross-patient seizure detection and SNNs. On one hand, we aim to explore how to improve cross-patient seizure detection performance from the perspective of utilizing EEG's biological characteristics and reducing potential energy. We apply biologically plausible SNNs for seizure detection to better capture the biological and temporal information of EEG signals. On the other hand, SNNs show a lacking performance compared with ANNs in existing tasks. We aim to explore suitable tasks and find real-world applications and practical scenarios of SNNs.

There are several challenges to applying spiking neural networks to epileptic signals. Firstly, the time dimension in EEG signals is fuzzy, as there are many ways to split and assign the time dimension in EEG signals to the time steps of SNNs. How we should design the time dimension in input data to better correspond to the spiking neural network is an important problem. Secondly, it is also important to design a suitable model architecture and select appropriate time steps to make full use of the channel information of EEG signals and the time series information of each channel so that the spiking neural network can capture and extract more features of EEG signals. Finally, there are many training methods for SNNs, however, the most suitable training method for practical biological data still needs to be explored.

In this paper, we demonstrate how spiking neural networks can achieve high performance in difficult cross-patient seizure detection settings, exceeding existing state-of-the-art ANN methods. We introduce a recurrent spiking neural network EESNN (EEG-based recurrent convolutional spiking neural network) that is composed of spiking neurons simulating the firing and signal propagation processes in real brain neurons and evaluate the performance of different time settings as well as architectures combined with training methods. Experiments considering both performance and theoretical energy estimation show the superiority of our model. In brief, our contributions are summarized as follows:

• We introduce a bio-inspired spiking recurrent neural network with a proper training method, which can achieve comparable state-of-the-art performance in cross-patient seizure detection. This can provide a better seizure detection model with more consideration of the biological properties of EEG and also broaden real-world application scenarios of SNNs.
• We extensively evaluated the cross-patient seizure detection performance of different SNN structures, training methods, and time settings, which has built a solid basis for understanding and evaluation of SNNs in the seizure detection task. We found that proper SNNs show a more superior performance than ANNs, indicating the potential abilities of SNNs for biomedical signal tasks.
• We have shown that our EESNN model can improve the theoretical energy efficiency by several orders of magnitude lower computational cost than ANNs. The result shows that our method has the potential to construct an energy-saving and efficient seizure detection system with neuromorphic computing.

## 2 Related work

There are two lines of research related to our work: automatic seizure detection and spiking neural networks.

### 2.1 Automatic seizure detection

Automatic epilepsy seizure detection based on EEG signals has attracted widespread attention. The mainstream seizure detection methods are based on deep neural networks due to their high accuracy and end-to-end computation. Commonly used network architectures include the convolutional neural network (Hu et al., 2018; Wei et al., 2019; Abiyev et al., 2020; O'Shea et al., 2020; Ke et al., 2021; Shen et al., 2023), recurrent neural network (RNN; Abdelhameed et al., 2018; Hu et al., 2020), graph neural network (Wang et al., 2020; He et al., 2022; Tang et al., 2022), Transformer (Ke et al., 2022; Sun et al., 2022), and their combination (Abdelhameed et al., 2018; Jia et al., 2020; Ke et al., 2022). However, these kinds of networks treat EEG signals as image-liked inputs, which may not better utilize biological information. There are also some works using spiking neural networks for epileptic seizure detection due to biological plausibility (Ghosh-Dastidara and Adeli, 2007; Ghosh-Dastidar and Adeli, 2009) and energy efficiency (Zarrin et al., 2020; Shan et al., 2023; Yang et al., 2023), however, the performance remains lacking compared with ANNs.

In automatic seizure detection tasks, the main challenge lies in the cross-patient setting, which focuses on the generalization ability for unseen patients that is essential to clinical application. Cross-patient detection does not work well for vanilla deep learning methods, and more strategies are required. Some works have used data augmentation methods (Wei et al., 2019; Gómez et al., 2020; Peng et al., 2022) to improve the accuracy of cross-patient detection. Another effective cross-patient epilepsy detection method is to use feature disentanglement to separate patient personality features and common epilepsy features (Zhang et al., 2020; Zhao et al., 2022). Some other methods applied meta-learning for the cross-patient problem, such as MUPS (Meta Update Strategy; Zhu et al., 2020) and MLCL (meta-learning on constrained transfer learning; Duan et al., 2020). Some works have also used domain adaptation (He and Wu, 2020; Nasiri and Clifford, 2021; Xia et al., 2022) or domain generalization (Ayodele et al., 2020) with multiple datasets to improve the model's generalization ability. Our work mainly explores spiking neural networks for cross-patient seizure detection, which are orthogonal to these methods. In this paper, we consider combining the cross-patient methods from our previous work (Zhang et al., 2023), including the data augmentation and adversarial strategy (see Section 3 for details).

### 2.2 Spiking neural networks

As the third generation of neural networks (Maass, 1997), SNNs have gained increasing attention recently due to their inherent energy-efficient computation (Lee et al., 2016) and efficient brain modeling (Kasabov, 2014). SNNs are applied in numerous fields, including computer vision (Xiao et al., 2021; Xiao M. et al., 2022; Niu et al., 2023), speech recognition (Wu et al., 2020; Auge et al., 2021), natural language processing (Xiao R. et al., 2022), brain modeling (application; Gütig, 2016; Sahu and Dash, 2023), etc. However, the performance of SNNs remains limited compared with ANNs, and the training of SNNs is much harder due to the non-differentiable spiking neuron model. Researchers have made lots of efforts to improve SNNs from both the network structure (Gu et al., 2020; Lotfi Rezaabad and Vishwanath, 2020; Comşa et al., 2021; Fang et al., 2021a; Kamata et al., 2022; Zhang et al., 2022) and the training method (Kim and Panda, 2021; Li et al., 2021; Perez-Nieves and Goodman, 2021; Xiao et al., 2021, 2023; Xiao M. et al., 2022). However, SNNs still fail to beat their ANN counterparts in performance. It is worth noting that most of the existing comparative experiments are conducted on tasks where ANNs perform well, such as computer vision, and many SNN model architectures are also based on existing ANN structures. So, it is essential to explore what kind of tasks SNNs are suitable for and what kind of SNN can better play on its unique advantages. Our work explores the novel application of SNNs on cross-patient seizure detection and broadens the real-world application of SNNs with better performance.

There are also some works that use spiking neural networks for epilepsy tasks. Zarrin et al. (2020) used feedforward spiking convolutional neural network for intracranial electroencephalography (iEEG) seizure detection under patient-specific setting, while Burelo et al. (2022) aimed at detecting epileptic high-frequency oscillations, using a fully connected feedforward spiking neural network under a patient-specific setting. Yang et al. (2023) applied a spiking convLSTM model for epilepsy seizure detection. However, these works are different from our settings, data/tasks, or model architecture. In particular, our work focuses on the cross-patient (patient-independent) setting, and we compared the performance of EESNN with various SNN architectures in Section 4.2.2 and found that our architecture has better performance.

## 3 Methods

In this section, we elaborate on two aspects of our method to solve the cross-patient seizure detection problem: the first is about SNN architecture with training methods, and the second is about other cross-patient algorithms except SNN.

### 3.1 SNN architecture and training methods

#### 3.1.1 Leaky integrate-and-fire neuron

Spiking neurons are inspired by biological neurons in the human brain, which is different from artificial neural networks. The difference between SNNs and ANNs mainly lies in two properties. Firstly, the spiking neuron uses a differential equation to maintain membrane potential and integrates the input signal. When the membrane potential reaches the threshold, it sends out a binary spiking signal. Secondly, the temporal binary spike train is used for information propagation between the spiking neurons, and the input and output of the neurons are both spike trains.

The leaky integrate and fire (LIF) model is the commonly used spiking neuron model. The dynamic of the membrane potential is described as shown in Equation (1):


(1) $$\begin{array}{ll} \tau_{m}\frac{du}{dt}=-\left(u-u_{reset}\right)+R\cdot I\left(t\right), & u<V_{th} \end{array}$$

where *u* represents the membrane potential, *I* represents the input current, *v*_*th*_ represents the firing threshold, and *R* and τ_*m*_ represent the resistance and leakage terms, respectively. When *u* reaches *v*_*th*_ at time *t*_*f*_, the neuron fires a spike and resets the membrane potential *u* to *u*_*reset*_, which is often set to 0. The spike train emitted by a neuron can be represented by the Dirac function $s\left(t\right)=\underset{t_{f}}{\sum }\delta \left(t-t_{f}\right)$. In practice, we simulate spiking neurons with discretization. Neurons are connected by weights *w*, and we consider the simple current model $I_{j}\left[t\right]=\underset{i}{\sum }w_{ij}s_{i}\left[t\right]+b_{j}$. The discrete computational form is described as shown in Equation (2):


(2) $$\{\begin{array}{l} u_{j}[t+0.5]=\lambda u_{j}[t]+\sum_{i}w_{ij}s_{i}[t]+b_{j},\\ s_{j}[t+1]=ℋ(u_{j}[t+1]-V_{th}),\\ u_{j}[t+1]=u_{j}[t+0.5]-V_{th}s_{j}[t+1], \end{array}$$

Where H(x) is the Heaviside step function, i.e., the non-differentiable spiking function, *s*_*i*_[*t*] is the binary spike train of neuron *i*, and λ is a leaky term related to the constant τ_*m*_ and discretization time interval for the LIF model. We use subtraction as the soft reset.

#### 3.1.2 EEG-based recurrent convolutional spiking neural network

**Figure 3:**
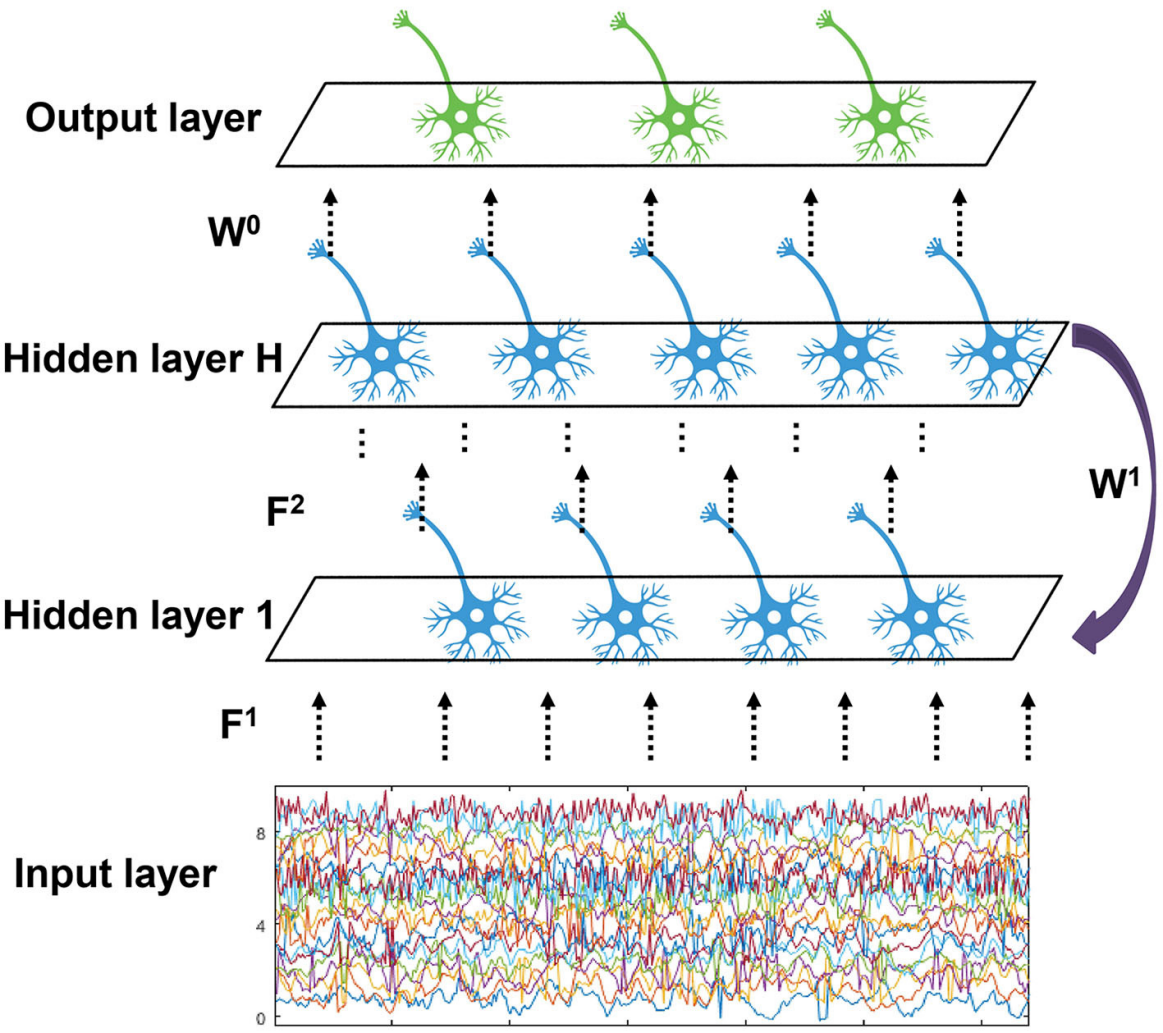
The structure of EESNN network. The network is composed of *H* hidden layers with feedforward connection weights *F*^1^, ..., *F*^*H*^, and there exists a feedback connection from the top hidden layer to the bottom with weight *W*^1^. The output layer reads the last hidden layer with weight *W*^0^.

We propose an EESNN model based on SNNs to automatically detect the seizures, as shown in Figure 3. EEG signals can be treated as an input current for SNNs (Zhang and Li, 2020; Xiao et al., 2021). There are hidden layers that consist of LIF neurons in an EESNN. Compared with common feedforward networks, the EESNN model adds a feedback connection from the last hidden layer to the first layer. Such kind of recurrence can better leverage temporal information from previous time steps at the network level, apart from the neuron level of SNNs, which may better handle time series data. Feedback connections are also shown beneficial to various tasks in previous works (Xiao et al., 2021; Yin et al., 2021; Kim et al., 2022; Xiao M. et al., 2022).

Finally, an output layer with readout neurons will perform classification. Formally, the inputs are connected to the first hidden layer with weight *F*^1^, the (*l*−1)-th layer is connected to the *l*-th layer with weights *F*^*l*^, and the last hidden layer is connected to the first hidden layer with weight *W*^1^. Let *u*^*l*^(*t*) and *s*^*l*^(*t*) be the output of *l*-th layer and *x*(*t*) be the input. This paper uses *H* groups of different spiking neurons to form the corresponding *H* layer. The discrete updating equation of model membrane potential is described as shown in Equation (3):


(3) $$\begin{array}{l} \{\begin{array}{l} u^{1}[t+1]=\lambda u^{1}[t]+W^{1}s^{H}[t]+F^{1}x[t]+b^{1}-V_{th}s^{1}[t+1],\\ u^{l+1}[t+1]=\lambda u^{l+1}[t]+F^{l+1}s^{l}[t+1]+b^{l+1}-V_{th}s^{l+1}[t+1],l=1,2,\cdots ,H-1. \end{array} \end{array}$$

In addition, an EESNN uses a two-dimensional convolutional structure (i.e., the linear operations *W*^1^ and *F*^*i*^ are convolutions), which is also adopted by many deep learning methods for seizure detection. Specifically, the input is formulated in a similar form to images, where the two dimensions correspond to the time dimension of an EEG and the electrode channels, and the “channel” in the context of images is 1. The convolution will perform transformations on both the spatial and temporal information of EEG signals. When it comes to the setting of SNNs, we should consider the additional temporal dimension of SNNs. There are two ways to consider the time. First, we can map the time dimension of EEG signals (i.e., the product of time window size and frequency) to one dimension in convolutional operations as introduced above, considering the time steps of SNNs as a separate dimension, with constant replicated inputs at each time step. Second, we may split the time of EEG signals as *T* = *t*_1_×*t*_2_, where *t*_1_ represents a small time slide and *t*_2_ represents a global number of time slides, and map each time slide to one dimension in convolutional operations while setting the time steps of SNNs as *t*_2_. We present the results of different time settings in Section 4.

For the final classification, the output layer of an EESNN is composed of one neuron, and we assume that it will not spike or reset (different from hidden neurons) but use the accumulated membrane potential to perform classification. The membrane potential will go through a sigmoid function to obtain the probability of whether it is a seizure. We classify it as a seizure if this probability is >50%, i.e., it is more likely to be a seizure than a non-seizure, which is commonly used for deep learning methods. During inference, this can also be simplified that if the accumulated membrane potential is positive, then it is classified as seizure.

#### 3.1.3 SNN training methods

##### 3.1.3.1 Implicit differentiation on the equilibrium state (IDE) method

We first consider the Implicit Differentiation on the Equilibrium state (IDE) method (Xiao et al., 2021) for SNN training. It decouples the forward and backward computational graphs, where the forward seeks to find the equilibrium state, and the backward seeks to find the implicit differentiation and gradient of the equilibrium state equation. Therefore, common SNN training problems can be avoided, such as non-differentiability during forward calculation, large memory overhead caused by storage of computational graph for backpropagation, etc.

The equilibrium state of the multi-layer EESNN with *u*_*reset*_ can be described as follows:

**Proposition 1**. (Xiao et al., 2021): If the weighted average inputs $\hat{x}\left[t\right]=\frac{\overset{t}{\underset{\tau =1}{\sum }}\lambda^{t-\tau }x\left[\tau \right]}{\overset{t}{\underset{\tau =1}{\sum }}\lambda^{t-\tau }}$ converge to an equilibrium point $\hat{x}\left[t\right]\to x^{*}$, and there exists γ ≤ 1 such that $||W^{1}|{|}_{2}||F^{N}|{|}_{2}\cdots ||F^{2}|{|}^{2}\leq \gamma ||V_{th}-u_{reset}||\leq \gamma {\left(V_{th}-u_{reset}\right)}^{N}$, then the weighted average spiking probability of multi-layer FSNN with discrete LIF model $\alpha \left[t\right]=\frac{\overset{t}{\underset{\tau =1}{\sum }}\lambda^{t-\tau }s\left[\tau \right]}{\overset{t}{\underset{\tau =1}{\sum }}\lambda^{t-\tau }}$ will converge to equilibrium points α^*l*^[*t*] → α^l*^, which satisfy the fixed-point equations $\alpha^{1*}=f_{1}\left(f_{n}•\cdots •f_{2}\left(\alpha^{1*},x^{*}\right)\right)$ and $\alpha^{l+1*}=f_{l+1}\left(\alpha^{l*}\right)$, where $f_{1}\left(\alpha ,x\right)=\sigma \left(\frac{1}{V_{th}-u_{reset}}\left(W^{1}\alpha +F^{1}x+b^{1}\right)\right)$, $f_{l}\left(\alpha \right)=\sigma \left(\frac{1}{V_{th}-u_{reset}}\left(F^{l}\alpha +b^{l}\right)\right)$, and σ(*x*) = min(1, max(0, *x*)).

Let $\alpha^{*}=f_{\theta }\left(\alpha^{*}\right)$ denote the fixed point equation of equilibrium state with the EESNN network parameter θ. If we take the derivative of θ on both sides of the equation, we can get the implicit differentiation (Bai et al., 2019): $\left(I-\frac{\partial f_{\theta }\left(a^{*}\right)}{\partial a^{*}}\right)\frac{da^{*}}{d\theta }=\frac{\partial f_{\theta }\left(a^{*}\right)}{\partial \theta }.$ According to the chain rule, the gradient of the loss function with respect to the neural network parameters is as follows:


$$\begin{array}{l} \frac{\partial L\left(a^{*}\right)}{\partial \theta }=\frac{\partial L\left(a^{*}\right)}{\partial a^{*}}\frac{\partial a^{*}}{\partial f_{\theta }\left(a^{*}\right)}\frac{\partial f_{\theta }\left(a^{*}\right)}{\partial \theta }=-\frac{\partial L\left(a^{*}\right)}{\partial a^{*}}\left(J_{g_{\theta }}^{-1}{|}_{a^{*}}\right)\frac{\partial f_{\theta }\left(a^{*}\right)}{\partial \theta }, \end{array}$$

where *g*_θ_(*a*) = *f*_θ_(*a*)−*a*, $J_{g_{\theta }}^{-1}{|}_{a^{*}}$ is the inverse Jacobian of *g*_θ_ evaluated at α^*^. This calculation of the inverse Jacobian can be solved effectively by the linear equations: $\left(J_{g_{\theta }}^{T}{|}_{{\text{a}}^{*}}\right)\text{x}+{\left(\frac{\partial L\left({\text{a}}^{*}\right)}{\partial {\text{a}}^{*}}\right)}^{T}=0,$ where *T* means the transpose operation. We can use Broyden's method (Bai et al., 2019; Xiao et al., 2021) or approximation method with acceleration (Fung et al., 2022) to solve the equation.

##### 3.1.3.2 Surrogate gradient method

There are other successful training methods for SNNs, and we also consider the surrogate gradient (SG) method. In SNNs, the binary spike train makes the back-propagation process non-differentiable. To solve the difficult training of spiking neural networks, researchers usually use the surrogate gradient methods (Wu et al., 2018; Fang et al., 2021a; Deng et al., 2022) to replace the non-differentiable terms with the derivative of a smooth function. Specifically, the non-differentiable term $\frac{\partial s}{\partial u}$ can be replaced by derivatives of piece-wise linear, sigmoid, or atan functions, whose expressions are: $h_{1}\left(u\right)=\frac{1}{a_{1}}sign\left(|u-V_{th}|\leq \frac{a_{1}}{2}\right),h_{2}\left(u\right)=\frac{1}{a_{2}}\frac{exp\left(\left(V_{th}-u\right)/a_{2}\right)}{{\left[1+exp\left(\left(V_{th}-u\right)/a_{2}\right)\right]}^{2}},h_{3}\left(u\right)=\frac{a_{3}}{2\left(1+{\left(\frac{\pi }{2}a_{3}x\right)}^{2}\right)},$ where *a*_1_, *a*_2_, *a*_3_ are hyperparameters.

SG is usually combined with the Backpropagation Through Time (BPTT) framework (Werbos, 1990), which is an extension of backpropagation to the temporal dimension. The gradients are iteratively calculated based on backpropagation from both spatial and temporal dimensions (Wu et al., 2018). There are also methods that improve BPTT for temporally online training. For example, online training through time (OTTT; Xiao M. et al., 2022) avoids the drawback of BPTT to backpropagate through previous time by tracking presynaptic traces of neurons so that gradients can be online calculated at each time and can also archive competitive performance. We will consider these methods with their commonly adopted network structures as well as our model in the experiments.

### 3.2 Other cross-patient algorithms

As introduced previously, there are several cross-patient algorithms proposing techniques to improve deep learning methods, which are orthogonal to network structures. We considered combining our SNN model with the data augmentation and adversarial strategy from our previous work (Zhang et al., 2023). We briefly introduce them, and all experiments include these techniques by default.

#### 3.2.1 EEG data augmentation

As there is only a small sample size of seizure periods leading to the class imbalance problem in real seizure datasets, EEG data augmentation is an important technique for cross-patient performance. Existing EEG data augmentation only considers the temporal characteristics (Wei et al., 2019; Gómez et al., 2020; Peng et al., 2022) and does not make good use of spatial information. Our previous work (Zhang et al., 2023) designed the spatio-temporal EEG augmentation (STEA) for the training data, which can achieve better performance than previous works. For any *t*-second EEG signals *x* with *c* electrode channels, we calculated the mean and variance matrix of the flattened vectors $\hat{x}\in R^{t\times c}$ and generated new EEG signals through the multi-gaussian distribution. We augmented training seizure data using STEA, which can largely alleviate the class imbalance problem.

#### 3.2.2 Adversarial strategy

Adversarial strategy (Zhang et al., 2023) aims at refining feature extraction by minimizing individual characteristics so that common features across different people are obtained for better cross-patient generalization. Specifically, an adversarial patient identity classifier will be added to the network, and with the alternative training between the epilepsy detection model and the identity discriminator under the adversarial objective (i.e., the discriminator is encouraged to classify identities while the model is encouraged to confuse the discriminator), the end-to-end feature extractor can well detect the seizure periods while it cannot distinguish the patient's identity. This training strategy can make the feature extractor automatically extract the individual-invariant features associated with epilepsy for cross-patient improvement. After training, the neural network model can extract common features among different patients, that is, patients cannot be distinguished based on features. This patient-invariant representation can be better generalized for unseen patients, improving the cross-patient performance of the model. We leveraged this adversarial identity classifier and training strategy for our EESNN model.

### 3.3 Overall pipeline

**Figure 4:**
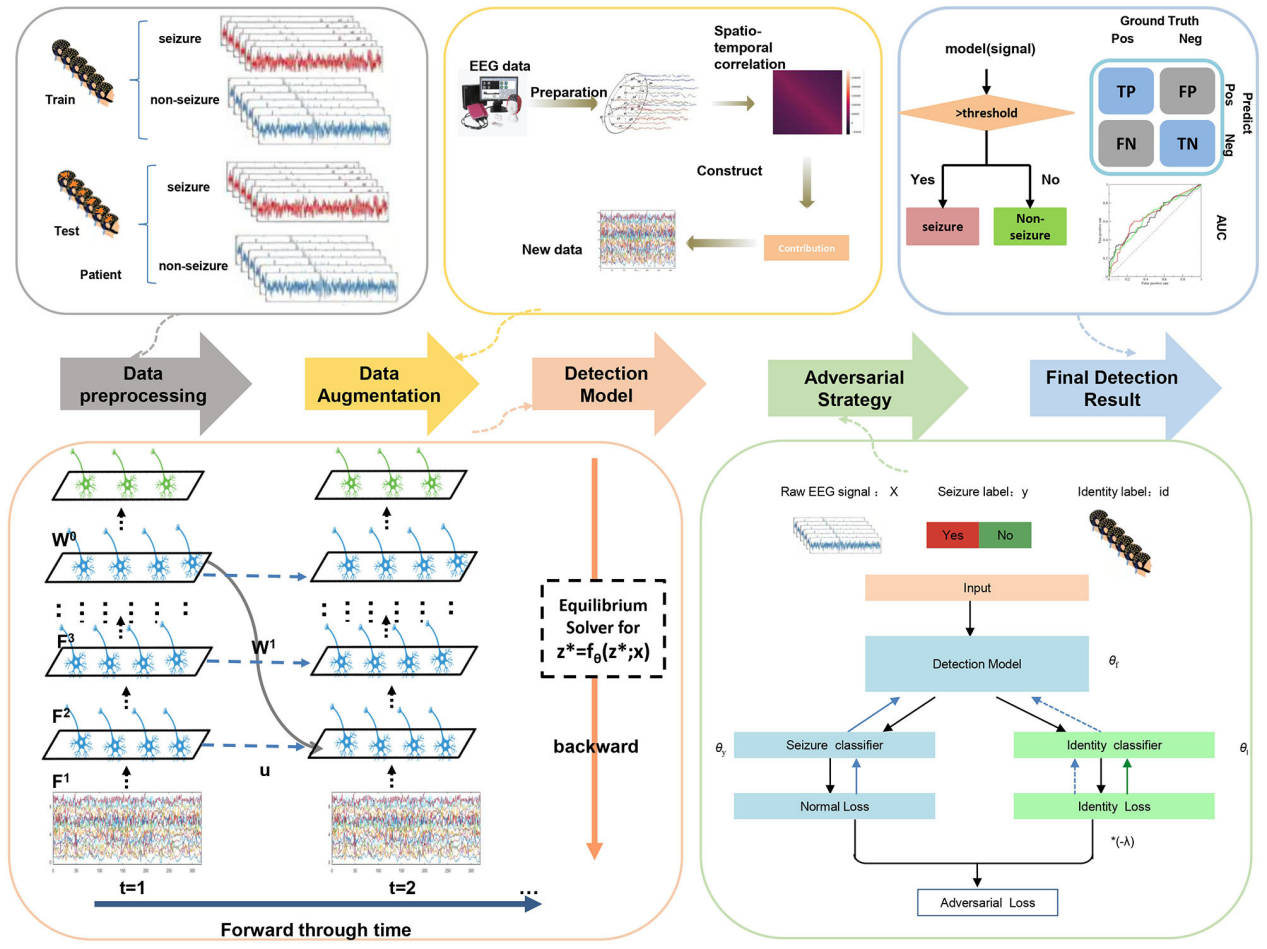
The overall framework of our methods.

The overall pipeline of our cross-patient seizure detection method is as follows: we first conduct Spatial-Temporal-EEG-Augmentation (STEA) on the EEG signals of the training set (Section 4). Then, EEG signals are fed as current input into spiking recurrent convolutional neural network EESNN (Section 3.1.2) with SNN training methods (Section 3.1.3) and adversarial strategy (Section 3.2.2) for seizure-invariant feature extraction and classification. The illustration of the overall method can be found in Figure 4.

## 4 Experiments

### 4.1 Experimental setup

Seizure detection aims to distinguish whether the EEG in a short time interval is in the ictal stage, so it is formulated as a binary classification task. After splitting the whole EEG signals into short segments with the same window size (*t* seconds), we get the EEG segments and denote the available data as (**x**_*i*_, **y**_*i*_), *i* = 1, ⋯ , *N*, where *N* is the number of segments and **y**_*i*_∈{0, 1} is a class label, with **y**_*i*_ = 1 corresponding to a seizure period and **y**_*i*_ = 0 corresponding to a non-seizure period. The EEG signal of the *i*-th sample is denoted as ${\text{x}}_{i}\in {\mathbb{R}}^{T\times C}$ where *C* is the channel dimension and *T* is the temporary dimension. The research goal is to design a classifier to correctly distinguish whether the patient is on seizure according to *t*-second EEG signal duration.

Specifically, we focus on cross-patient seizure detection in this paper. Under the basic problem of seizure detection, the cross-patient setting has a sample of *M* patients, where *M*_*D*_ patients are for model training and other *M*_*T*_ patients are for model testing, with *M*_*D*_+*M*_*T*_ = *M*. The value of *M* for different datasets is shown in Table 1, and the value of *M*_*T*_ is specified in the experiments, i.e., the test patient number in Tables. Cross-patient settings are the data pattern of actual medical treatment. Besides, cross-patient seizure detection can be reproducible, and generalizable to future patients.

**Table 1:** The statistical information of two seizure datasets used in this work.

**Datasets**	**No. of subjects**	**EEG channels**	**Place method**	**Sampling frequency (Hz)**	**Seizure (h)**	**Non seizure (h)**	**Total record (h)**
CHB-MIT	23	21	International 10–20 system	256	3.26	964.59	967.85
PKU1st	19	19	International 10–20 system	500	0.99	72.18	73.17

In our experiments, except for the general setting, we also consider “fine-tuning” settings. Since there are few patients in the test set, we divide a small amount of data to fine-tune the model for better generalization. This might also correspond to some real-world situations when patients come for treatment for the second time and doctors can collect their data. “Fine-tuning” settings can also verify the flexibility of the model to adapt to new patients. In the experiments below, we consider both settings. The latter is marked as “fine-tuning” in the results; other results belong to the former setting by default.

For concrete experiments, we chose one public epilepsy dataset, CHB-MIT (Shoeb and Guttag, 2010), and one clinical epilepsy dataset, PKU1st. The CHB-MIT dataset collected by Boston Children's Hospital includes 23 patients' EEG data with a 256 Hz sampling frequency and 23 channels. It is the most commonly used public dataset for EEG detection. The PKU1st dataset is the latest EEG data collected by the Department of Pediatrics, Peking University First Hospital, and approved by the Ethics Committee of the Peking University First Hospital (2021-225). The PKU1st dataset consists of EEG signals from 19 patients, the EEG sampling frequency is 500 Hz, and there are 19 channels in EEG signals. The details of the two datasets can be found in Table 1. More details can be found in the Supplementary material.

There are several steps to preprocess raw EEG data before training. Firstly, we conduct EEG data cleaning to remove duplicate channels and invalid data. Secondly, we downsample EEG signals to a lower frequency to reduce noise and memory usage. Specifically, we downsample 256 to 64 Hz and downsample 500 to 50 Hz. Thirdly, we split the continuous EEG into many short-time segments with ground-truth expert labels and set the ratio of seizure and non-seizure segments number to be 1:5. The EEG window length is usually selected between 2 and 12 s arbitrarily.

In the comparison experiment, we keep the evaluation setting the same as the compared method. Under the leave-one-out setting, we employ *N*-fold cross-validation to partition the EEG segments into training and testing sets, where *N* is the patient number of a dataset. Thus, it can better measure the overall capability of our model.

In our experiments, four statistical indicators are used for the performance evaluation of the proposed method. Some indicators are defined as shown in Equations (4–7):


(4) $$\begin{array}{l} Sensitivity=\frac{TP}{TP+FN}, \end{array}$$

(5) $$\begin{array}{l} Specificity=\frac{TN}{TN+FP}, \end{array}$$

(6) $$\begin{array}{l} RAccuracy=\frac{r\cdot TP+TN}{r\cdot \left(TP+FN\right)+TN+FP}, \end{array}$$

(7) $$\begin{array}{l} GMean=\sqrt{Sensitivity\times Specificity}. \end{array}$$

We also use AUC (the area under the receiver operating curve) as one of the metrics. In clinical practice, the most concerned indicator is Sensitivity. In addition, the balance of sensitivity and specificity is also necessary, which can be reflected in the GMean and RAccuracy.

Cross-patient seizure detection aims to classify the seizure and non-seizure periods. The goal of this work is to build a reliable and accurate seizure detection method to facilitate and accomplish the diagnosis of epilepsy.

### 4.2 Results

#### 4.2.1 A representative example for cross-patient seizure detection using our SNN

To illustrate the experiment setting and how the EESNN facilitates well-behaved generalization ability, let us consider a one-hidden-layer EESNN to detect seizure abnormalities in the cross-patient setting. We use the PKU1st dataset and split the EEG signals into many 2-s segments labeled as a seizure or not.

##### 4.2.1.1 Training

For any 2-s EEG signal in a training set with 10 patients, the time step of EESNN is set as 2. The input EEG signals is reshaped into three-dimensional (3D) tensors (electrode channels × time samples × 1).

##### 4.2.1.2 Inference (test)

The trained EESNN model is used to detect the seizure period for another nine patients in the testing dataset which has no overlap with training data.

##### 4.2.1.3 Our method outperforms state-of-the-art ANN methods

We compare the single-layer EESNN model with the existing state-of-the-art ANN model (Ke et al., 2021; Zhang et al., 2023), and the two methods are trained under the same setting. The specific experimental results are shown in Table 2. Our spiking neural network with only one layer still has good performance in the cross-patient epilepsy detection task and exceeds the artificial neural network. More comprehensive comparison experiments can be seen in Section 4.2.3.

**Table 2:** Comparison of performance between the state-of-the-art artificial neural network and our spiking neural network in the cross-patient epilepsy detection task (2-s, 9-person PKU1st dataset without fine-tuning) under the same training setting.

**Network type**	**Sensitivity (%)**	**AUC (%)**	**GMean (%)**	**Raccuracy (%)**
ANN	56.89	72.91	66.58	67.40
SNN	**80.82**	**78.04**	**69.89**	**70.63**

Bold means the better performance between ANN and SNN.

#### 4.2.2 Evaluation of different SNN architectures, training methods, and time settings

In this section, we demonstrate how SNNs can be applied to cross-patient seizure detection effectively. Although in the previous section, SNNs showed a potentially superior brain abnormality detection ability compared to ANNs, not all SNNs perform well. This section will explore how to effectively use SNNs to process brain signals.

**Figure 5:**
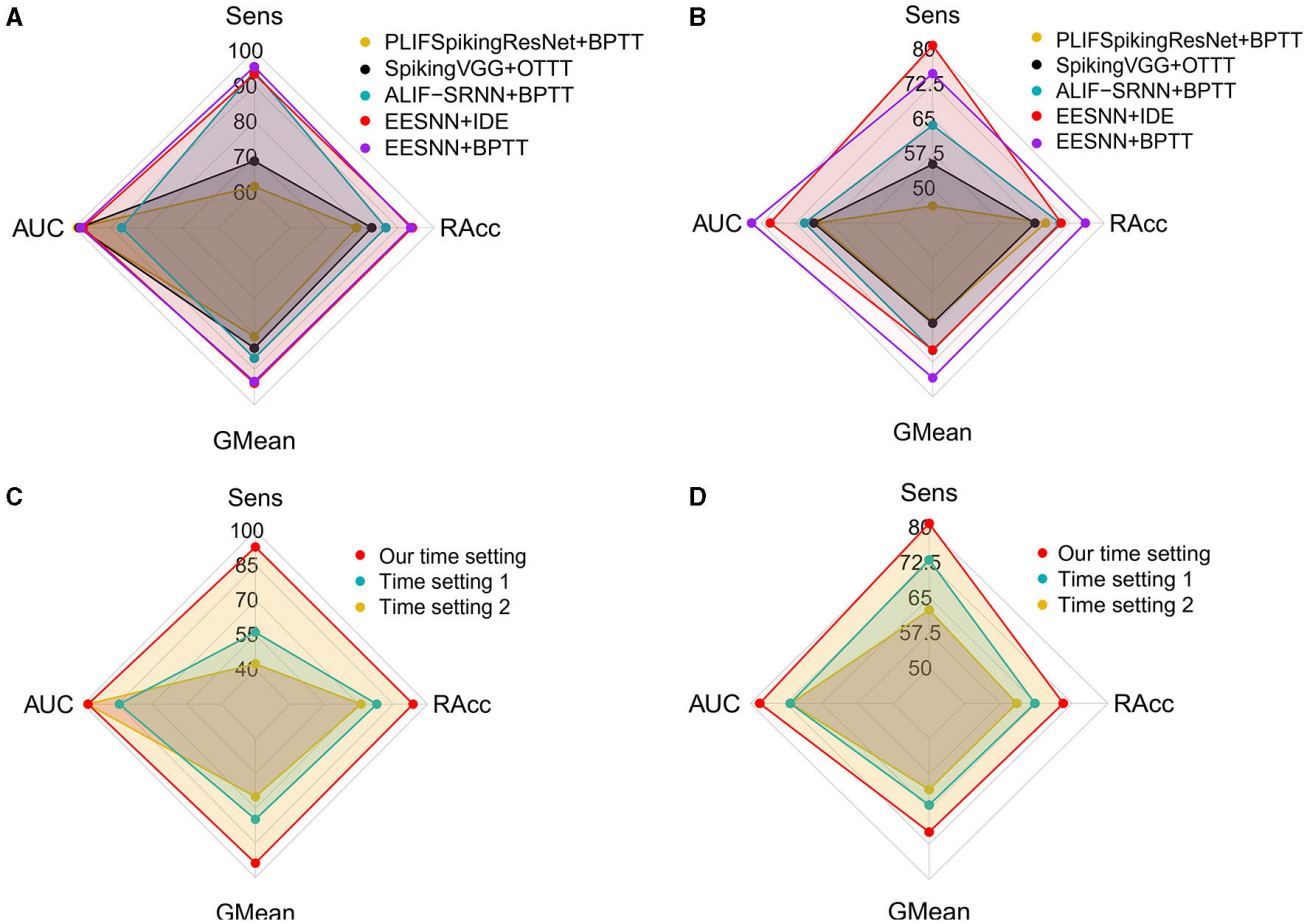
Evaluation of different spiking neural networks, training methods, and time correspondences. **(A, C)** Used the CHB-MIT dataset under a 4 s time window size with one test patient. **(B, D)** Used the PKU1st dataset under a 2 s time window size with nine test patients.

We first analyzed the performance of different time settings. We compared several time settings in EEG signals. The first method maps the time dimension of EEG signals (i.e., the product of time window size and frequency) to one dimension in convolutional operations and considers the time steps of SNNs as a separate dimension, with constant replicated inputs at each time step, and we set time steps to be 12. In the second method, we split the time of EEG signals as *T* = *t*_1_ × *t*_2_, where *t*_1_ represents a small time slide and *t*_2_ represents a global number of time slides, and mapped each time slide to one dimension in convolutional operations while setting the time steps of SNNs as *t*_2_. The third method maps the time dimension of EEG signals to one dimension in convolutional operations and considers the time steps of SNNs as a separate dimension that is set to be 2, which is the time setting in our method. As Figure 5 shows, the third approach can achieve the best performance. Compared with the first setting, our method has superior performance, probably because fewer time steps can introduce noise to increase generalization ability. In the seizure detection task, a larger time step of the SNN does not guarantee a better result.

Additionally, for the SNN architectures and training methods, we compare several baseline models, including ALIF RSNN (Yin et al., 2021) trained with BPTT, PLIFSpikingResNet (Fang et al., 2021b) trained with BPTT, and SpikingVGG (Simonyan and Zisserman, 2015) trained with online training through time (OTTT; Xiao M. et al., 2022), with our method. The best performance across all time settings is reported for all models. As Figure 5 shows, EESNN trained with IDE (Xiao et al., 2021) achieves the best performance.

#### 4.2.3 Performance comparisons and analyses

To demonstrate the superior performance of our approach, we also compare the performance with other popular cross-patient seizure detection methods. We compare our method to several baseline models, including MIDS+WGAN (Wei et al., 2019), First Seizures model (Gómez et al., 2020), SDG (Ayodele et al., 2020), CW-SRNet (Ke et al., 2021), Dist-DCRNN (Tang et al., 2022), ConvLSTM (Yang et al., 2022), and Dense CNN (Saab et al., 2020). Additionally, during comparison, we kept experimental settings consistent with theirs.

The brief descriptions of the 10 baseline models are as follows:

• **MIDS+WGAN** (Wei et al., 2019) starts with MIDS (the merger of the increasing and decreasing sequences) data prepossessing and WGAN (Wasserstein Generative Adversarial Nets) data augmentation and then employs a 15-layer CNN architecture for cross-patient detection with the standard training procedure.
• **AAN** (Zhang et al., 2023) uses the STEA data augmentation and PANN training strategy, which is the same as our method, and then employs a 16-layer CW-SRNet for cross-patient detection.
• **IBA** (Zhao et al., 2022) is a kind of multi-view learning method with feature disentanglement; each EEG sample has a window size of 1 s with 50% overlapping, and it uses two GAN models for cross-patient seizure detection under standard training procedure.
• **Dist-DCRNN** (Tang et al., 2022) is a diffusion convolutional recurrent neural network that can model the spatiotemporal dependencies in EEGs. We reproduced the model with STEA data augmentation and a PANN training strategy, which are the same as our method.
• **LSTMSNN** (Yang et al., 2023) applies a spiking convLSTM model after using a sliding window of 1 s with 50% overlap to crop the EEG signals with AdamW optimizer under standard training procedure.
• **ConvLSTM** (Yang et al., 2022) uses the convolutional long short-term memory network for cross-patient seizure detection. We reproduced it on the same dataset under the cross-patient setting with the standard training procedure.
• **First Seizures model** (Gómez et al., 2020) implements a fully convolutional network (FCN) with a time-shift between consecutive windows of 1/4 s for the seizure period of the dataset and regularization strategies.
• **SDG** (Ayodele et al., 2020) uses the technique of supervised domain generalization with additional much more datasets for training. The backbone model is a CNN architecture for feature extraction followed by an LSTM layer for seizure detection.
• **CW-SRNet** (Ke et al., 2021) exploits a custom CNN architecture composed of CW-Block with attention mechanism and SE-Block. CW-SRNet is a **non-cross-patient** (patient-specific) model with the state-of-the-art performance. We reproduced it on the same dataset under the cross-patient setting under standard training procedure.
• **Dense CNN** (Saab et al., 2020) exploits densely connected inception network trained by imperfect but plentiful archived annotations. We reproduced it on the same dataset under the cross-patient setting under standard training procedure.

Tables 3, 4 show the performance comparisons between our method and various methods. Compared with AAN (Zhang et al., 2023), which was the previous state-of-the-art method and which uses the same training setting as our model, our method can improve the performance probably because our spiking recurrent neural network can capture the biological information in EEG. Moreover, compared with other machine learning methods, our approach can achieve superior performance due to the effective network structure, training method, adversarial strategy, and special data augmentation. In particular, our EESNN model achieves the best performance under the fine-tuning setting. The method in this paper significantly improves the performance of cross-patient epilepsy detection.

**Table 3:** The comparison with existing cross-patient seizure methods.

**Dataset**	**Method**	**Time window size**	**Test patient number**	**Sensitivity (%)**	**AUC (%)**	**GMean (%)**	**RAccuracy (%)**
CHB-MIT	First Seizures Model (Gómez et al., 2020)^†^	4	1	78.48	–	88.53	89.18
	SDG (Ayodele et al., 2020)^†^	–	–	71.45	-	73.69	73.73
	AAN (Zhang et al., 2023)^*^	4	1	**95.71**	97.98	94.13	94.15
	EESNN (ours)^*^	4	1	91.67	**98.64**	**94.38**	**94.42**
	LSTMSNN (Yang et al., 2023)^†^	12	1	-	97.5	–	–
	EESNN (ours)^*^	12	1	**86.29**	**99.52**	**92.15**	**92.35**
	CW-SRNet (Ke et al., 2021)	2	9	43.33	79.55	64.91	70.29
	AAN (Zhang et al., 2023)^*^	2	9	61.31	**88.23**	**74.58**	**76.02**
	EESNN (ours)^*^	2	9	**65.70**	79.21	71.15	71.38
	IBA (Zhao et al., 2022)^†^	1	1	77.78	93.61	81.79	83.31
	AAN (Zhang et al., 2023)^*^	1	1	**92.43**	93.80	**85.71**	**86.40**
	EESNN (ours)^*^	1	1	83.04	**94.11**	82.79	83.98
	MIDS+WGAN (Wei et al., 2019)^†^	5	1	74.08	–	82.76	83.27
	CW-SRNet (Ke et al., 2021)	5	1	42.67	96.18	64.97	70.08
	AAN (Zhang et al., 2023)^*^	5	1	**99.74**	**97.56**	**93.54**	**93.73**
	EESNN (ours)^*^	5	1	94.34	95.66	92.79	92.81
PKU1st	CW-SRNet (Ke et al., 2021)	5	1	2.91	62.75	16.58	48.71
	ConvLSTM (Yang et al., 2022)	5	1	3.9	36.10	16.58	37.2
	Dense CNN (Saab et al., 2020)	5	1	23.3	79.7	47.71	60.5
	Dist-DCRNN (Tang et al., 2022)^*^	5	1	67.0	**87.1**	77.18	77.95
	AAN (Zhang et al., 2023)^*^	5	1	**83.50**	86.59	**83.11**	**83.11**
	EESNN (ours)^*^	5	1	81.55	84.20	82.32	82.32
	CW-SRNet (Ke et al., 2021)	2	9	30.62	76.59	54.52	63.85
	ConvLSTM (Yang et al., 2022)	2	9	22.97	50.66	42.59	50.98
	Dense CNN (Saab et al., 2020)	2	9	41.63	64.98	58.27	61.6
	Dist-DCRNN (Tang et al., 2022)^*^	2	9	47.8	70.8	62.61	64.9
	AAN (Zhang et al., 2023)^*^	2	9	56.89	72.91	66.58	67.40
	EESNN (ours)^*^	2	9	**80.82**	**78.04**	**69.89**	**70.625**

^*^Means that these models' results are under the same STEA data augmentation and PANN strategy as ours. ^†^Means that these models' results are derived directly from their papers, and other results are reproduced by us with the standard training procedure. Bold means the best performance among different models.

**Table 4:** The comparison with previous methods under the same training procedure and the finetune setting.

**Dataset**	**Method**	**Time window size**	**Test patient number**	**Sensitivity (%)**	**AUC (%)**	**GMean (%)**	**RAccuracy (%)**
CHB-MIT	AAN+finetune (Zhang et al., 2023)	5	1	95.83	94.15	90.80	90.94
	EESNN+finetune (ours)	5	1	**98.40**	**99.85**	**98.81**	**98.82**
	AAN+finetune (Zhang et al., 2023)	4	1	99.37	98.23	87.32	88.05
	EESNN+finetune (ours)	4	1	**99.99**	**99.87**	**97.80**	**97.83**
	AAN+finetune (Zhang et al., 2023)	2	9	**90.38**	90.95	81.04	81.53
	EESNN+finetune (ours)	2	9	84.95	**96.64**	**90.60**	**90.80**
PKU1st	AAN+finetune (Zhang et al., 2023)	5	1	**81.93**	87.97	82.43	82.44
	EESNN+finetune (ours)	5	1	78.31	**98.74**	**87.66**	**88.22**
	AAN+finetune (Zhang et al., 2023)	2	9	82.24	84.99	**76.06**	**76.29**
	EESNN+finetune (ours)	2	9	**88.01**	**86.14**	73.63	74 81

Bold means the better performance between ANN (AAN model) and SNN (EESNN model) under the finetune setting.

**Figure 6:**
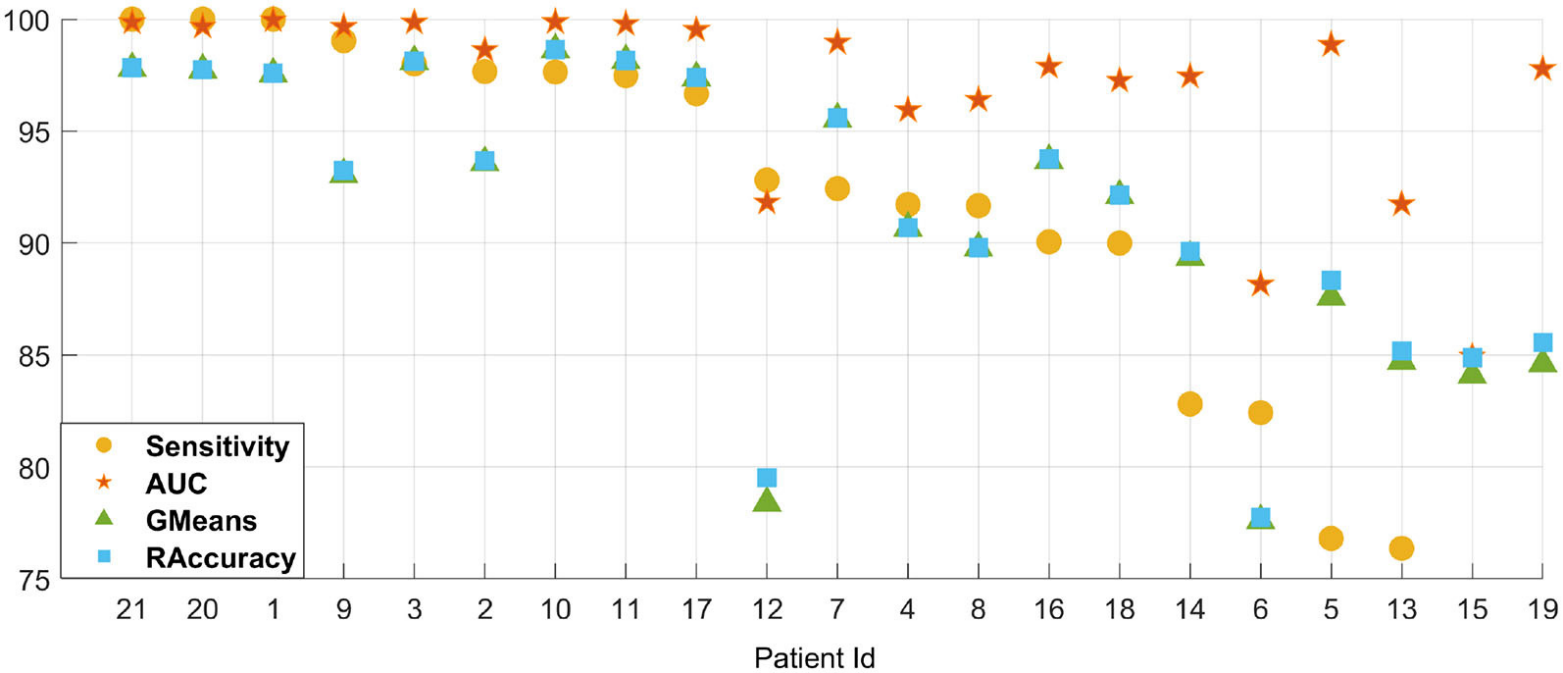
The leave-one-out (LOO) result of cross-patient detection on the CHB-MIT dataset. Patient IDs indicated on the X-axis are sorted by sensitivity.

In addition, the leave-one-out result of cross-patient detection on the CHB-MIT dataset is shown in Figure 6, where we select each patient alone as the testing set and other patients for training. Among all patients, we have an average of 90.46% sensitivity, 96.86% AUC, 91.44% GMean, and 91.68% RAccuracy under 4 s EEG segments with a finetune setting. The single result of each patient is shown in Figure 6 after sorting by sensitivity, which indicates the powerful generalization ability of our method.

#### 4.2.4 Theoretical energy estimation

As for efficient neuromorphic computation, the energy and computational costs are important statistics. We calculated the average spiking probability per time step of our trained model for inference [i.e., the total spike counts/(the number of neurons * time steps)] and compared the theoretical energy estimation following previous works (Yin et al., 2021; Wu et al., 2021)citepwu2021tandem,yin2021accurate with the state-of-the-art ANN model to demonstrate the advantage of our SNN. For ANNs, every synaptic operation requires a MAC (multiplication and accumulation) operation, while for event-driven SNNs, it only requires an accumulate (AC) operation when a spike is triggered. According to the 45 nm CMOS processor (Horowitz, 2014), the energy for 32 bit FP MAC operation is 4.6 pJ and for AC operation it is 0.9 pJ, and we calculated the theoretical estimation of energy cost for different models based on it. As shown in Table 5, the average spiking probability is only around 0.5%, indicating the sparsity of spikes as well as small operation numbers and energy consumption. We calculated the corresponding operation numbers for all synapses of the neuron population and the energy consumption (i.e., the operation number multiplied by energy for each one) in Table 6. The results show that our EESNN model has several orders of magnitude lower computation cost and energy consumption than ANN models, which is beneficial for building an energy-efficient system.

**Table 5:** The average spiking probability in EESNNs per time step for all neurons.

**Layer**	**Layer 1**	**Layer 2**	**Layer 3**	**Layer 4**	**Layer 5**	**Average**
Spiking probability	0.0018	0.0048	0.0054	0.0060	0.0062	0.0052

Our model is trained on the CHB-MIT dataset with 2 s window size and nine test patients.

**Table 6:** Comparison of computation cost and energy cost of different methods under CHB-MIT dataset with a 2 s window size and nine test patients, where RNN is the corresponding ANN network to EESNN, and the AAN model (Zhang et al., 2023) is the state-of-the-art network under cross-patient seizure detection.

**Network type**	**Operation number**	**Energy consumption (mJ)**	**Average spiking probability**
EESNN	**1.4 × 10^8^**	**1.4 × 10^−1^**	0.0052
RNN	2.6 × 10^10^	1.2 × 10^2^	1
AAN (Zhang et al., 2023)	1.6 × 10^9^	7.2 × 10^0^	1

Bold means the fewest computation cost and energy cost.

#### 4.2.5 Explanation experiments

**Figure 7:**
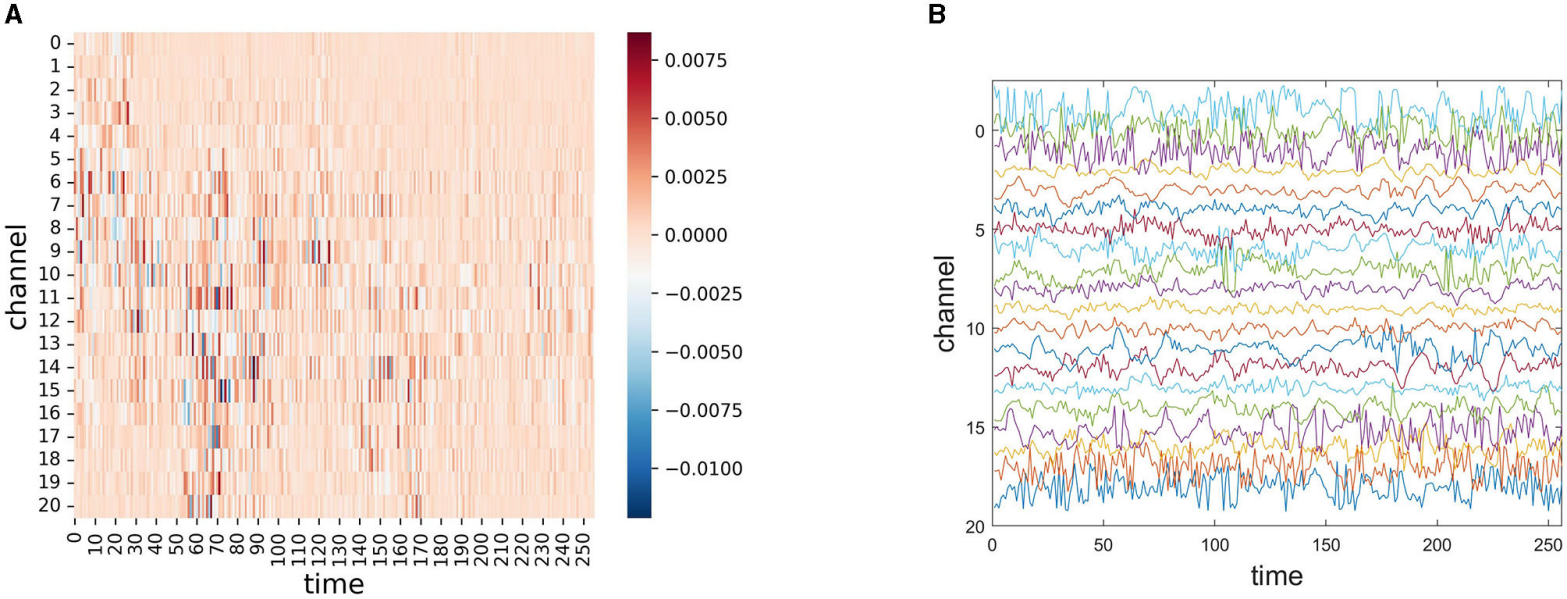
**(A)** Gradient-based localization of one 4 s seizure EEG signal segment through EESNN, where the x-axis represents different channels, and the y-axis represents different times. The important channels and temporal moments of each EEG signal segment are dark colors. **(B)** The corresponding raw EEG signal where different colors represent different channels.

To explore the interpretability of our model, we performed an interpretable visualization experiment of EESNN through the Grad-CAM technique (Selvaraju et al., 2017). Grad-CAM can visually locate the important areas of the input that influence the classification result of the model most via gradients, and it can be used to show how our model infers a seizure. Figure 7A shows the importance of each channel and each moment in seizure EEG signals for seizure detection by our model, which is the gradient of each position considering the classification output of the model. The red color represents positive gradients, and the blue color represents negative gradients. A darker color means a larger absolute magnitude of the gradient, which indicates the importance of this position identified by our model. As the EEG signal uses a 4-s period with 64 HZ and 21 channels, the GramCAM result in Figure 7A has 256 × 21 units. In Figure 7B, we show the corresponding raw EEG signal in the CHB-MIT dataset, where the channel order is consistent with Figure 7A, and different colors represent different channels. This method may help to discover some structures of the EEG data that indicate a seizure. Additionally, we would like to mention that our work is mainly aimed at assisting clinical diagnosis and does not intend to replace explainable treatment. It may serve as a timely warning, which can relieve the pressure on doctors.

## 5 Conclusion

This article introduces a brain-inspired spiking neural network for cross-patient seizure detection. Our proposed spike neural network structure EESNN may better capture the bio-characteristics of EEG signals and imitate the spiking signal processing in real brain neurons, enabling performance improvement with low energy costs. Moreover, this paper explores how the model architecture, time correspondence, and training method can make the spiking neural network more suitable for EEG data processing.

Our work has found that brain-inspired SNNs can outperform ANNs in epileptic seizure detection, especially showing better generalization ability under the cross-patient experiment and finetune setting experiment, which indicates that SNNs may be better suitable for brain activity tasks. In existing works, SNNs usually have a lacking performance compared to ANNs since they mainly focus on computer vision tasks, while brain activity data may be a better fit for SNNs. On the other hand, SNNs can be implemented on dedicated hardware with high capacity and low energy consumption; thus, our work has the potential to build an accurate hardware-friendly, low-power neuromorphic system.

## Data availability statement

Publicly available datasets were analyzed in this study. This data can be found at: https://archive.physionet.org/physiobank/database/chbmit/ and https://github.com/snowbbbb/EESNN-epileptic-seizure-detection.

## Ethics statement

The studies involving humans were approved by the Ethics Committee of the Peking University First Hospital (2021-225). The studies were conducted in accordance with the local legislation and institutional requirements. Written informed consent for participation was not required from the participants or the participants' legal guardians/next of kin in accordance with the national legislation and institutional requirements.

## Author contributions

ZZ: Formal analysis, Investigation, Methodology, Software, Validation, Visualization, Writing - original draft. MX: Formal analysis, Methodology, Writing - review & editing, Software. TJ: Data curation, Resources, Writing - review & editing, Funding acquisition. YJ: Data curation, Funding acquisition, Supervision, Writing - review & editing. TL: Conceptualization, Funding acquisition, Supervision, Writing - review & editing. XZ: Conceptualization, Funding acquisition, Resources, Supervision, Writing - review & editing. ZL: Conceptualization, Funding acquisition, Resources, Supervision, Writing - review & editing.
