# Supplementary material for: Efficient and generalizable cross-patient epileptic seizure detection through a spiking neural network
Source: Front Neurosci. 2024 Jan 10;17:1303564. doi: 10.3389/fnins.2023.1303564 (PMC10805904; doi:10.3389/fnins.2023.1303564)
Supplement: Supplementary file 1 [file Data_Sheet_1.PDF]

Supplementary Material

1 IMPLEMENTATION DETAILS

The model details of EESNN including kernel size, stride, padding and channel can be found in Table S1.

In experiments, we implement our methods by PyTorch and train our models by the Adam optimizer (Kingma and Ba, 2015) with batch size 8 for 50 epochs. The learning rate is set to be a constant value during training, which is obtained by grid search in the range of 10^{-8} to 10^{-2} at a log-uniform interval to get the best learning rate based on the validation set.

Table S1. The multi-layer EESNN model details.

layer id	input channel	output channel	basic operation	kernel size	stride	padding
Input layer	1	64	2D Convolution	5	(1,1)	2
Hidden Layer 1	64	256	2D Convolution	5	(1,1)	2
Hidden Layer 2	256	384	2D Convolution	5	(2,1)	2
Hidden Layer 3	384	384	2D Convolution	5	(1,1)	2
Hidden Layer 4	384	256	2D Convolution	5	(1,1)	2
Feedback layer	256	64	ConvTranspose	3	(2,1)	1
Output layer	256	2	Fully connected	-	-	-
Max layer	2	1	Max	-	-	-

2 MORE RESULT DETAILS

The detailed experimental results of different spiking neural network architectures and time correspondences are presented in Tables S2 and S3.

Our operation number is for the entire neuron population, which is calculated by considering all synaptic operations for all neurons. Specifically, they are calculated as:

asp = the total spike counts / (the number of neurons * time steps).

operation number = $\sum_i$ (asp of layer i) * (the number of neurons in layer i) * (induced synaptic operations for each neuron in layer i) * time step,

3 MORE DATASET DETAILS

The details of each patient in the PKU1st dataset are shown in Table S5. The details of each patient in the CHB-MIT dataset are shown in Table S4. Both two datasets are composed of EEG signals of children with epilepsy. The PKU1st dataset has a more balanced gender ratio than the CHBMIT dataset.

4 TRAINING DYNAMICS

The training epochs of EESNN are fixed for 50 epochs. Figure S1 presents the dynamics of training accuracy and AUC of EESNN, showing that our model has good and stable training dynamics. Our model can achieve nearly 100% training accuracy and training AUC after training.

Table S2. Evaluation of different spiking neural networks.

dataset	method	time window size	test patient number	Sensitivity	AUC	GMean	RAccuracy
CHB-MIT	PLIFSpikingResNet+BPTT	4	1	61.62%	98.66%	84.01%	82.62%
	SpikingVGG+OTTT	4	1	68.81%	99.15%	80.74%	78.44%
	ALIF-SRNN+BPTT	4	1	76.52%	84.11%	84.11%	83.77%
	EESNN+BPTT	4	1	**95.45%**	**98.32%**	93.47%	93.50%
	EESNN+IDE	4	1	92.93%	97.89%	**93.70%**	**93.70%**
PKU1st	PLIFSpikingResNet+BPTT	2	9	46.19%	67.68%	63.83%	67.20%
	SpikingVGG+OTTT	2	9	55.19%	68.49%	64.13%	64.86%
	ALIF-SRNN+BPTT	2	9	63.59%	70.35%	70.02%	70.35%
	EESNN+BPTT	2	9	74.73%	**82.12%**	**75.89%**	**75.9%**
	EESNN+IDE	2	9	**80.82**%	78.04%	69.89%	70.63%

Table S3. Evaluation of different time correspondences.

dataset	method	time window size	test patient number	Sensitivity	AUC	GMean	RAccuracy
CHB-MIT	Time setting 1	4	1	56.06%	84.26%	74.78%	77.91%
	Time setting 2	4	1	42.42%	**98.09%**	65.02%	71.04%
	Our setting	4	1	**92.93%**	97.89%	**93.70%**	**93.70%**
PKU1st	Time setting 1	2	9	73.07%	71.65%	64.14%	64.68%
	Time setting 2	2	9	62.39%	71.53%	60.85%	60.88%
	Our setting	2	9	**80.82%**	**78.04%**	**69.89%**	**70.63%**

Table S4. The CHB-MIT dataset details, where “F” and “M” represent “Female” and “Male” respectively.

Patient id	Sex	Age	Seizure time(s)	Record Time(s)	Seizure number
1	F	11	449	40.55	7
2	M	11	175	25.3	3
3	F	14	409	28	7
4	M	22	382	155.9	4
5	F	7	563	39	5
6	F	1.5	147	66.7	9
7	F	14.5	328	68.1	3
8	M	3.5	924	20	5
9	F	10	280	67.8	4
10	M	3	454	50	7
11	F	12	809	34.8	3
12	F	2	1515	23.7	21
13	F	3	547	33	12
14	F	9	117	26	8
15	M	16	2012	40	20
16	F	7	94	19	10
17	F	12	296	21	3
18	F	18	323	36	6
19	F	19	239	30	3
20	F	6	302	29	8
21	F	13	203	33	4
22	F	9	207	31	3
23	F	6	431	28	7
24	-	-	527	22	16

REFERENCES

Kingma, D. P. and Ba, J. (2015). Adam: A method for stochastic optimization. In *International Conference on Learning Representations*

Table S5. The PKU1st dataset details, where "F" and "M" represent "Female" and "Male" respectively.

Patient id	Sex	Age	Seizure time(s)	Record Time(s)	Seizure number
1	M	14.5	548	11088	2
2	M	1	167	16041	1
3	M	7	219	11701	2
4	F	10	566	11605	2
5	F	5	55	29333	2
6	F	8.5	429	11721	3
7	F	7	95	12007	3
8	F	5	164	10193	2
9	F	4.5	104	8745	1
10	M	2	47	10488	2
11	M	10	73	16106	2
12	M	6.5	214	11680	3
13	M	10	26	10859	2
14	F	8	83	10986	2
15	F	5	176	32853	4
16	M	11	205	10800	2
17	F	1.5	46	11071	2
18	F	6	233	10732	2
19	M	4	108	15407	1

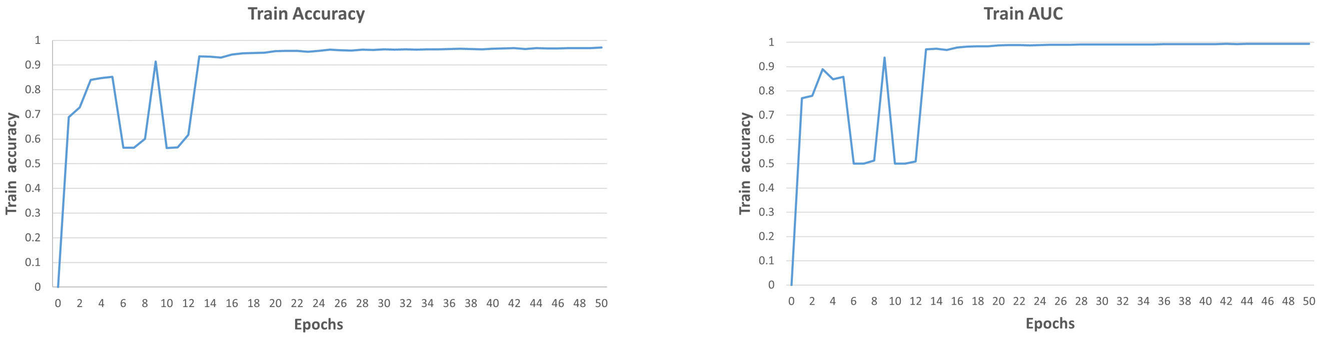

(a)

(b)

Figure S1. The training dynamics of EESNN.
